# City of Hope Quality of Life-Ostomy Questionnaire Validity and Reliability Assessment on a Croatian Sample

**DOI:** 10.3390/ijerph17030768

**Published:** 2020-01-25

**Authors:** Vesna Konjevoda, Marko Zelić, Radenka Munjas Samarin, Davorina Petek

**Affiliations:** 1 Department of Public Health, Faculty of Medicine, University of Ljubljana, Poljanski nasip 58, Ljubljana 1000, Slovenia; 2 University of Applied Health Sciences, Mlinarska cesta 38, Zagreb 10 000, Croatia; 3 Department of Surgery, Clinical Hospita l”Sveti Duh”, Sveti duh 64, Zagreb 10 000, Croatia; 4 Department of Surgery, University Hospital Rijeka, Krešimirova ul.42, Rijeka 51 000, Croatia; marko.zelic@uniri.hr; 5 Department of Surgery, Faculty of Medicine, University of Rijeka, Ul. Braće Brancetta 20/1, Rijeka 51 000, Croatia; 6 Faculty of Health Studies, University of Rijeka, Viktora Cara Emina 5, Rijeka 51 000, Croatia; 7 Radenka Munjas Samarin, SmartUp Healthcare Market Research, Savska cesta 22, Zagreb 10 000, Croatia; radenka.munjas@ri.t-com.hr; 8 Department of Family Medicine, Faculty of Medicine, University of Ljubljana, Poljanski nasip 58, Ljubljana 1000, Slovenia; Davorina.petek@gmail.com

**Keywords:** validation, CoH-QoL-OQ, ostomy, quality of life, HRQoL

## Abstract

The aim of this study was to validate City of Hope Quality of Life-Ostomy Questionnaire (CoH-QoL-OQ) for assessing the quality of life (QoL) of ostomy patients in the Republic of Croatia. The CoH-QoL-OQ is widely used, but has not been translated or validated so it can be used in the Republic of Croatia. This cross-sectional study encompassed 302 surgery patients with colostomy, ileostomy, or urostomy (182 (60.3%) male and 120 (39.7%) female), whose average age is 59 (M = 59.3, SD = 15.8). The CoH-QoL-OQ was translated into Croatian language using accepted guidelines for translation. Patients were recruited in a telephone conversation, followed by mail containing the CoH-QoL–OQ delivered to the home addresses of the patients who agreed to participate. The collected data were analyzed to verify psychometric properties of the questionnaire on the Croatian sample. All subscales showed high level of internal consistency (Cronbach α = 0.73–0.89). The test-retest reliability indicated a very satisfactory temporal stability (r = 0.99). The Confirmatory Factor Analysis (CFA), showed that the originally established model was not adequate for the data (χ^2^ = 4237.88, *p* < 0.01, CFI = 0.540, NNFI = 0.481, RMSEA = 0.113). However, after modification that excluded problematic items, the data showed a better fit with the theoretical model (except for the LR chi-square test that remained statistically significant: χ^2^ = 1144.28, *p* < 0.01, CFI = 0.869, NNFI = 0.855 RMSEA = 0.077). We conclude that the CoH-QoL-OQ is a valid, reliable, and reducible instrument for measuring the health-related quality of life (HRQoL) among Croatian patients with ostomy in clinical research and clinical practice.

## 1. Introduction

According to data from relevant scientific literature, 700,000 Europeans have ostomy, while in the US there are about 750,000 patients suffering from this condition [1]. The main causes of ostomy surgeries include colorectal carcinoma (CRC), inflammatory bowel disease (IBD), trauma, acute diverticulitis, ileus, bladder cancer, and other diseases.

The second biggest public health problem of the population of the Republic of Croatia is cancer (2). In the Republic of Croatia, the incidence of colorectal, rectum, and anus cancer in 2015 was 17% in males and 12% in females [2]. In spite of the national preventative measures and programs, the trend of colorectal and rectal cancer together became the third most common cause of death in 2017, with an incidence of 16% in males and 13% in females [3]. Colorectal cancer is the second most common cause of death in men and women in Europe, with 446,000 newly diagnosed cases each year [4]. According to unofficial data, since there is no register or database of persons with ostomy in the Republic of Croatia, it is estimated that there are about 7500 persons suffering from this condition, which is about 0.2% of the population.

The quality of life (QoL) assessment in general is one of the significant outcome measures of major surgical procedures and treatments.

The health related quality of life (HRQoL) is a multi-dimensional concept commonly used to examine the impact of health status on the QoL [5]. Generic QoL instruments (subjective evaluation of one’s personal satisfaction with overall health and well-being), are not sensitive enough to detect the specific impact that ostomy has on ostomates’ QoL [6]. “With a multidimensional QOL instrument, focusing on the effects of an intestinal stoma, specific areas of concern of ostomates can be identified. They include physical well-being and symptoms, psychological well-being, social well-being and spiritual wellbeing.” [6] (page 127). 

According to relevant scientific literature, ostomy negatively affects the QoL of persons affected [5,6]. Among the most significant problems affecting their QoL is inadequate preoperative preparation, with emphasis on ostomy site marking and psychological support [7]. The acceptance of ostomy may be influenced by patient’s age, gender, the degree of involvement in decision-making, the cause of ostomy surgery, as well as whether ostomy is performed as an emergency or elective procedure and whether it is temporary or permanent [6,8].

Furthermore, it is associated with inability to control gases and sounds, constipation, tiredness and travel difficulties, change in style of clothing, and dissatisfaction with appearance [8,9]. Problems experienced by persons with ostomy also depend on the period of time after the surgery, so common stressors reported by patients during hospitalization included stoma formation, diagnosis of cancer, and preparation for self-care [10]. After discharge, the stressors experienced by patients were associated with body changes, altered sexuality, and impact on social life and activities [9,10].

According to the literature and previous research, psychosocial problems identified by the ostomy patients in the past 15 years as the most common are the following: poor body image perception and self-respect, depression, sexual difficulties, and lower psychosocial adaptation [11]. This has a negative impact on their self-esteem and HRQoL [11]. Regardless of the time passed since the stoma formation, obstacles to QoL fall in one of the three categories: ostomy-related concerns and impact on life, limitations in physical and social activities, and negative impact on physical and mental health [12]. The time passed since the surgery also has a major impact on the QoL of persons with ostomy [13].

“Detecting differences and changes among stoma patients requires the use of a more specific QoL instrument compared with instruments targeting the general population or patients with other conditions”, also confirms Wafaa Gameel et al. in 2010 [14] (p. 2).

By examining the scientific literature, it was established that there are only several questionnaires which evaluate HRQOL among ostomy patients: Stoma care QoL index, City of Hope-Quality of Life-Ostomy Questionnaire (CoH-QoL-OQ), Ostomy adjustment scale, and Ostomy adjustment inventory-23. The CoH-QoL-OQ is a comprehensive, multidimensional, self-report instrument designed to assess the QoL for individuals with intestinal ostomies. It is a condition-specific assessment instrument that consists of 90 questions divided into three sections: demographic and clinical characteristics, impact of stomas on lifestyle, and impact on the QoL. The last section consists of 43 questions arranged into four domains: physical well-being, psychological well-being, social well-being, and spiritual well-being, with responses scored from 0 to 10 [15]. The CoH-QoL-OQ was developed to assess the QoL not only of patients with carcinoma but also of patients with IBD, trauma, acute diverticulitis, ileus, bladder cancer, and other diseases that require ostomy [15]. To the best of our knowledge, so far, questionnaire validation has been done in the following languages: Chinese [15], Portuguese in Brazil [16], Persian [17], and Turkish [18]. CoH-QoL-OQ is widely used, therefore it would be very beneficial to use it when comparing the results. 

In the Republic of Croatia there is no specific validated questionnaire for assessing the QoL of ostomy patients. In the search of databases (i.e., PubMed, ScienceDirect, Medline) no scientific assessment of the QoL of persons with ostomy in the Republic of Croatia was found to date. One article based on the Croatian sample of 41 persons with ostomy [19], was published by the Southeastern European Medical Journal (SEEMEDJ).

By assessing the QoL, we will come to a better understanding of causes and consequences and will be able to take certain corrective actions in providing health care based on a holistic approach with the final aim of improving the physiological, psychological, emotional, and social levels of the QoL of persons with ostomy [20,21].

The aim of this study was to validate CoH-QoL-OQ for assessing the QoL of ostomy patients in the Republic of Croatia. 

## 2. Patients and Methods

The validation of the CoH-QoL-OQ was undertaken as part of a larger study on the subject of QoL of people with a stoma. Permission to conduct the study was obtained from ethics committees of four Croatian University Hospitals included in the study.

### 2.1. Methods Translation

The CoH-QoL-OQ was translated into Croatian language using accepted guidelines for translation, which are forward- and back-translations. The English version of the questionnaire was translated into Croatian by two native speakers of Croatian, fluent in English and versed in medical terminology. Two different translators independently translated the questionnaire back into English. The differences were minimal, and they were resolved by consensus. The back-translated English version was compared with the original English version to ensure that no loss of meaning or context occurred during the translation process.

### 2.2. Patients/Material

The validation process of the questionnaire was performed using the data about 302 patients with a stoma, 182 male and 120 female participants, from January to December 2018. The validation included surgery patients with colostomy, ileostomy, or urostomy, permanent or temporary, regardless of the indication and medical diagnosis (malignant disease, diverticulitis, IBD, ileus, intestinal injury, and bladder cancer), between the age of 18 and 84. The majority of patients were aged between 51 and 70, with the mean age of 59 (M = 59.3, SD = 15.8; male mean age M = 60.51, SD = 15.54, female M = 57.41, SD = 15.98). The largest number of respondents had a colostomy (221), followed by patients with ileostomy (75), while only six respondents had urostomy. A total number of 212 respondents had a permanent ostomy. A smaller sample of 50 patients was included in the verification of temporal stability of the scale. They filled out the scale twice with a two-week interval between measurements.

### 2.3. Instrument CoH-QoL-OQ

Grant and Davis [15,22], developed the CoH-QoL-OQ as an adult patient self-report instrument designed to assess QoL. Grant et al. (2004), published a review and psychometric testing of the CoH-QoL-OQ to assess the QoL of ostomy patients [23]. 

The CoH-QoL-OQ consists of 43 items. In addition to the overall CoH-QoL-OQ result, the items are categorized into four subscales: physical health (items 1–11), psychological (items 12–24), social (items 25–36), and spiritual well-being (items 37–43). 

All items are Likert scale questions with grades from 0 to 10. Reverse items are recorded prior to the analysis according to the authors’ instructions, so that zero represents the worst and 10 represents the best evaluation. Total subscale scores are calculated by adding scores of all scale items and then dividing the total score by the number of items in each subscale.

The authors of the CoH-QoL-OQ declared that request for permission was not necessary and that researchers may adapt the questionnaire as needed.

### 2.4. Data Collection

Participants were members of the Croatian Crohn’s and Ulcerative Colitis Association (HUCUK) and all currently active associations of patients with a stoma in Croatia nationwide. Data on patients and their contact details were obtained from the HUCUK and the Croatian Association of patients with stoma (ILCO). A permission to contact the patients was obtained from the competent persons in the associations. Patients signed the consent to participate in the study. After the purpose and aim of the research were presented to them in a telephone conversation, the CoH-QoL-OQ and consent forms to participate in the study translated into Croatian were delivered to their home addresses with return envelopes. A total of 580 questionnaires were distributed to patients, and 302 respondents returned the completed questionnaire, which was a response rate of 52%. Respondent data are shown in Table 1.

### 2.5. Statistical Analysis

Statistical analysis was performed using IBM SPSS Statistics version 25.0.0.125 and AMOS Version 22.0 [24]. The accepted *p* value was *p* < 0.05. Expected power of statistical tests for a sample of 302 patients is > 0.90.

Categorical variables were summarized as absolute and relative frequencies. For continuous variables median and interquartile range (1st and 3rd quartile) was shown. The Shapiro–Wilk test was used to test distribution normality. One-way ANOVA was used to verify if gender groups differ significantly in average scores of CoH-QoL-OQ (total and subscales).

Reliability of the CoH-QoL-OQ was measured in two ways; by calculating the test-retest correlations (Spearman’s Rank correlation coefficient between two points of measurement), and by calculating the internal consistency coefficient, Cronbach alpha (values of Cronbach alpha > 0.80 were considered as indicators of good internal consistency). 

The validity of the instrument was examined based on several indicators. Face validity, meaning that the instrument covers all relevant aspects of the measured phenomena, was based on medical experts’ opinions [17], and estimated by the degree of agreement on the validity of items between experts with long-term work experience with ostomy patients: one surgeon (an abdominal surgery specialist, a professor at a medical school), one nurse specialized in ostomy (a lecturer at the University of applied health sciences), one licensed enterostomal therapist (ET), with the certificate of the World Council of Enterostomal Therapists (WCET). They reviewed the instrument and agreed by consensus that the items were relevant and understandable to the respondents and covered all aspects of the QoL of persons with a stoma. 

The convergent and discriminant analyses were performed by measuring item-subscales correlation, following the rule that each subscale item should be correlated to a higher degree with their own subscale score, and with the scores of other subscales to a lower degree.

Since the CoH-QoL-OQ has been validated by the authors, only CFA was used to confirm a four-factor model stated by the authors in the Croatian sample of ostomy patients. 

## 3. Results

### 3.1. Reliability

The descriptive parameters of the individual subscales are shown in Table 2, as well as the internal consistency indicator Cronbach alpha.

All subscales show high level of internal consistency (0.73–0.89), and the total scale has the highest Cronbach alpha score (0.95), meaning that in the Croatian sample the CoH-QoL-OQ seems to be a reliable instrument in terms of item consistency. Regarding temporal consistency, all subscales and the total score show extremely high levels of correlation between two points of measurement, indicating very satisfactory temporal stability.

### 3.2. Age Differences in Average CoH-QoL-OQ Scales

**Figure 1:**
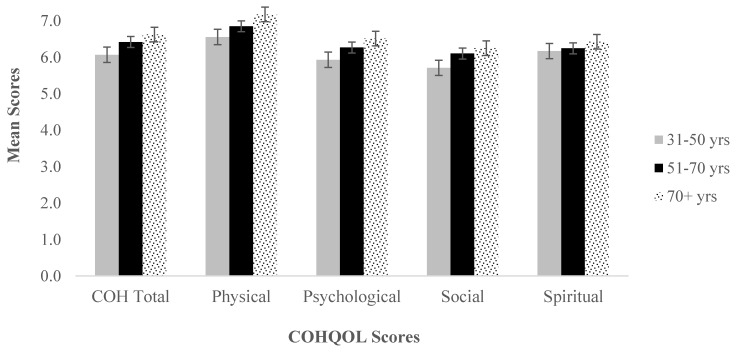
Average scores for total COH and subscales according to age groups.

Since our sample consisted of a majority of older patients, it was verified if age distribution had any impact on average scores of CoH-QoL-OQ. Results are shown in Figure 1.

Levene’s test of homogenity of variances shows no significant differences in variances between groups, so a series of one-way ANOVAs was calculated. The one-way ANOVA shows no statistically significant differences in average scores of COH scale (all *p* > 0.05).

### 3.3. Content Validity

In addition to the subscale level, the item-total correlation was calculated for individual items, each with the total score of its own subscale and with other subscales in order to verify the scale validity (Table 3).

If a commonly agreed criterion that the correlation of items with its own subscale should be >0.40 is adopted, then it is evident that on all three subscales, except Physical, there are items that do not meet the criterion of convergent validity. On the Psychological subscale, item 17 has the lowest correlation with the total score on the scale, on the Social subscale, items 31, 35, and 36 have very low convergent validity, and on the Spiritual, items 37 and 42 have low validity.

Regarding discriminant validity, the items should be in a lower correlation with the total scores of other subscales than with their own total score. On the Croatian sample, all subscales seem to meet this criterion. Additionally, the coefficients of correlation between the subscales are expected to be lower than the coefficients of the internal consistency for each subscale, which generally is the case here (Pearson coefficients ranging 0.40–0.80, all *p* < 0.01). The highest correlation was found between Social and Psychological subscales (0.80) and it is higher than Cronbach alpha for the Spiritual subscale (0.73).

In Table 4, each individual item’s correlation with total CoH-QoL-OQ and its own subscale is shown.

### 3.4. Confirmatory Factor Analysis (CFA)

As the last step of the validation of CoH-QoL-OQ, a Confirmatory Factor Analysis was performed to verify the four-factor model established and validated by the authors and used on the Croatian sample of patients. The overall fit refers to whether the covariance matrix proposed by the model deviates from the actually observed correlation matrix [25].

Since CFA does not allow for missing data, the data file was pre-arranged and 10 respondents with missing data were excluded from the analysis and the CFA was performed on a sample of *n* = 291.

**Figure 2:**
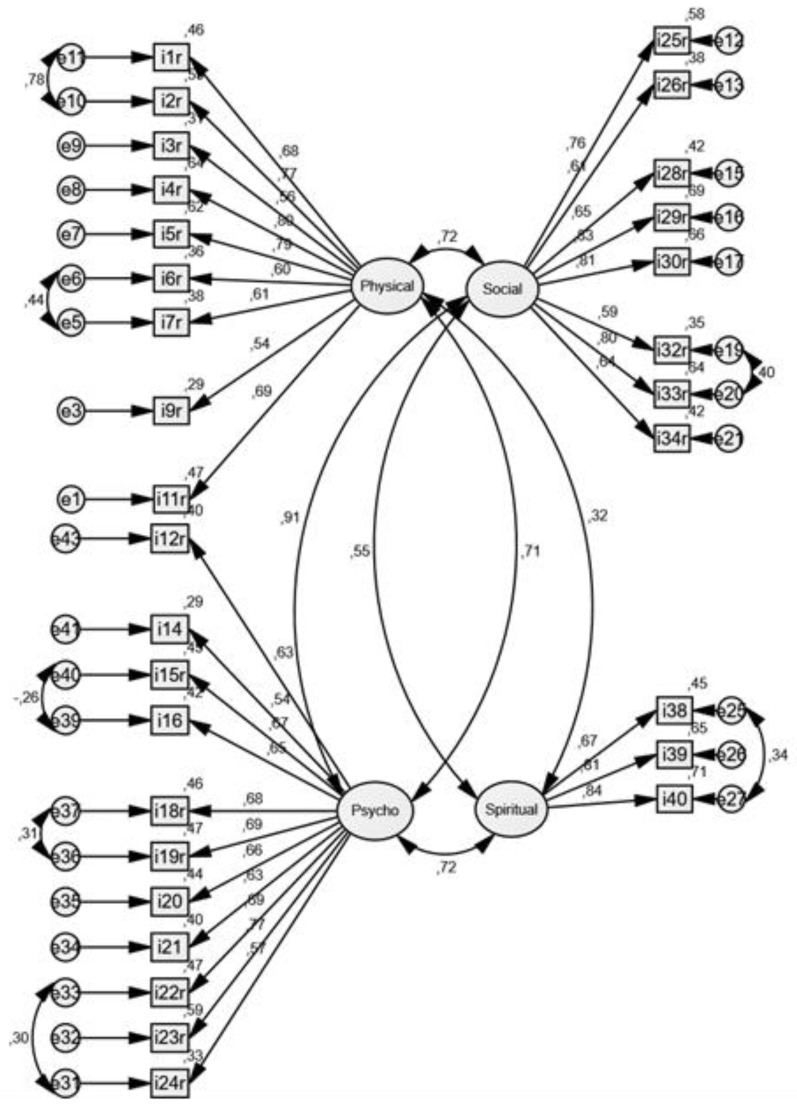
Confirmatory Factor Analysis of the Croatian version of CoH-QoL-OQ whose shortened version consisting of 31 items is a better model fit.

The initially established model was not adequate for the data: χ^2^ = 4237.88, *p* < 0.01, CFI = 0.540, NNFI = 0.481, RMSEA = 0.113. Therefore, several steps were taken to achieve a better fit of data and theoretical model. Firstly, we introduced covariances between error variances in the tested model where it was indicated by the modification indices (these indices in Amos program make suggestions on loosening some model parameters to improve the overall fit). Secondly, variables with standardized regression weights < 0.50 were removed from the model, assuming that they do not describe a relevant amount of variance of the common factor. Afterwards, the CFA model was finalized as shown in Figure 2.

The post-modification indicators show a relatively satisfactory fit (except for the LR chi-square test): (χ^2^ = 1144.28, *p* < 0.01, CFI = 0.869, NNFI = 0.855 RMSEA = 0.077), although the indicators are not ideal. Therefore, CFA suggests better model fit after removing some items. Namely, items 8 and 10 were removed from the Physical well-being factor, items 13 and 17 were removed from the Psychological well-being, items 27, 31, 35, and 36 were removed from the Social, while items 37, and 41–43 were removed from the Spiritual well-being. The shortened version consisted of 31 items.

## 4. Discussion 

The aim of this study was to verify if the Croatian version of CoH-QoL-OQ used in Croatian ostomy patients confirms reliability, validity, and factor structure of the original CoH-QoL-OQ developed by Grant and Davis [22], for measuring the QoL of patients with ostomy. By reviewing scientific papers Grant and Ferrell [23], found that a stoma, regardless of the reason for surgery (malignant disease or not), has an important impact on the QoL. They also confirm that the general QoL assessment tools are not sufficiently sensitive to detect a specific, ostomy-conditioned impact on the QoL [23]. In numerous studies [26,27,28], CoH-QoL-OQ was used as a survey instrument for assessing the HRQoL of persons with ostomy and proved to be a reliable survey instrument. No specific HRQoL questionnaire for assessing the QoL of ostomy patients has been translated or validated in the Republic of Croatia. Studies on the QoL of patients with ostomy in Croatia have not been conducted nor published to date. This study includes data collected from the entire Republic of Croatia, and it is also interesting because 34.5% of the respondents have had ostomy longer than 4 years, of which 21.9% longer than 8 years.

Based on Cronbach alpha coefficients calculated for each CoH-QoL-OQ subscale, and also the total score of 43 items, it can be concluded that all subscales and the total CoH-QoL-OQ show very satisfactory levels of internal consistency in the Croatian sample. Additionally, the temporal stability verified on a smaller subsample (*n* = 50) over a two-week period supports excellent test-retest reliability of all scales measured by CoH-QoL-OQ. Such results are consistent with those found in the literature [17], in which Cronbach alpha coefficients for all subscales were close to 0.70 or higher, as well as the results by Karaveli Cakir and Ozbayr [18], who found Cronbach alpha for the total CoH-QoL-OQ scale to be 0.95. It can be argued that an internal consistency above 0.90 stops being acceptable, as it indicates that some items do not contribute to the measured latent factor because of too high correlation with other items [29]. Our extremely high correlation might indicate that the period of retesting is too short for such stable construct as QoL. However, on a subscale level, coefficients indicate a single factor per subscale, as it was originally planned, which is useful in clinical practice.

In order to further verify the reliability and validity of CoH-QoL-OQ in this sample, the relationship between items was analyzed. It was expected that items belonging to the same underlying factor would be correlated, but too high correlation (>0.70), would suggest too much overlapping between items. The inter-item and item-total correlations in the Croatian sample show that items belonging to the same subscale correlate among them to a higher degree than they do with items of other subscales, but a more detailed investigation suggests that the subscale Physical well-being is the only subscale that completely matches the expected levels of correlation. In the other three subscales, there are some items that highly correlate with other subscales, or have low item-total correlation. For example, in the Psychological well-being scale, one item (item 24: Are you fearful that your disease will come back?), correlates relatively high (0.64) with the Social subscale, and one item (item 21: How satisfied are you with your appearance?) with the Spiritual subscale. The lowest internal consistency of the Spiritual subscale is in line with the finding that one item shows higher correlation with the Social (item 37: How much uncertainty do you feel about your future?), than it does with its own subscale. Taken together, it can be concluded that on the total subscale level, CoH-QoL-OQ shows good reliability and in general good convergent and discriminant validity, but some patterns of inter-correlations suggest there are questionable items that require further validation. This could be due to cultural differences; for example, being fearful that their disease will come back for ostomy patients in Croatia might have greater social implications, since they are dependent on others and it might change social functioning. 

Anaraki et al. [17], also evaluated the convergent and discriminant validity based on the correlations of items with the total scale and other scales, concluding that in the Iranian sample, the CoH-QoL-OQ among ostomy patients showed a satisfactory level of correlations, and that each subscale measured a single trait. When compared to our sample, it can be seen that the correlations of items with their own subscales ranged closely 0.40–0.70, and the correlation with other scales was equal to 0.03–0.50, which is a good psychometric property. However, in our sample some items showed less than ideal correlation with their own subscale (e.g., the Social well-being item 0.11, the Spiritual well-being item 0.20), and some were moderately to highly correlated to other subscales (the Social well-being item 0.70 with the Psychological)—suggesting that at least some subscales (like the above mentioned Spiritual well-being), could be measuring more than one specific trait. Spiritual items seem to imply a more complex construct than spiritual functioning than the authors originally intended. Perhaps Croatian society relates spirituality mostly to religious functioning, since it is mostly a Catholic society, without having deeper spiritual understanding and beliefs beyond specific religious behaviors such as praying. 

Even though there were no difficulties with the content validity [30], it can be concluded that some inter-item correlations are less than ideal and do not fully replicate results from other authors.

However, sufficient correlation with well-established measures of QoL (>0.70 but less than 0.90), as well as negative association with well-established measures of opposite constructs, such as depression or anxiety would be true evidence of convergent and divergent validity. This study is limited by lacking use of such measure because validation was done as a part of a larger study with repeated measures. Therefore, it was estimated that strain on patients with additional questionnaire length was not justified. Future studies should focus on investigating convergent and divergent validity of CoH-QoL-OQ. 

The final step of validation was performing CFA according to the original structure of 43 items, grouped into four subscales: Physical, Psychological, Social, and Spiritual well-being. The exploratory factor analysis was not performed since the main idea was to verify the existing model. The initial CFA model did not show good model fit, indicating that the covariance matrix proposed by the model deviates significantly from the matrix in this sample (χ^2^ = 4237.88, *p* < 0.01). Since the likelihood ratio of the chi-square test or the deviance test is sensitive to sample size, on samples larger than 200 the probability of achieving statistical significance (type II error) is greatly increased. In addition, its deficiency is linked to the exact fit, which is rare in social research, so we decided to use additional indicators: RMSEA (root mean square error of approximation), CFI (comparative fit index), NNFI (Bentler–Bonett non-normed fit index). All three indicators were consistent, showing poor data fit with the model (CFI = 0.540, NNFI = 0.481, RMSEA = 0.113). Since the previously calculated reliability and validity showed that the Croatian version of the CoH-QoL-OQ instrument showed satisfactory levels of reliability, and generally good validity with some problematic items, we used available methods to try to improve the model fit. We were most concerned about factor loadings < 0.50, indicating a small correlation between the observed variable and the corresponding common factor.

After introducing covariances between error variances and removing variables with small regression weights from the model, the model fit was improved to a more satisfactory level. Even though the LR of the chi-square still indicated that a null hypothesis of good fit with data should be rejected (χ^2^ = 1144.28, *p* < 0.01), other indicators showed improvement in fitting a factor structure to a four-factor model postulated by the original authors (CFI = 0.869, NNFI = 0.855 RMSEA = 0.077—CFI is very close to the level of 0.90, NNFI is much higher than before, but still below the ideal level of 0.95 cut-off, and RMSEA is a good fit < 0.08). The number of items was reduced in the final model. The final solution also shows a rather high correlation between the Social and Psychological factors, indicating an overlap in measurement, and suggests further validation and refinements of this instrument are needed. However, the internal consistency is improved for two subscales, the Social and the Spiritual, and now all subscales have Cronbach alpha score > 0.85, indicating very good reliability of results in each subscale of CoH-QoL-OQ.

Gao et al. [15], in their Chinese validation of CoH-QoL-OQ, used 32 items in the Confirmatory Factor Analysis. They were trying to validate their own results from the exploratory factor analysis, and such reduced model showed a good fit to a four-factor model, with satisfactory levels of scale reliabilities for all except the Spiritual well-being scale. It is possible that some cultural differences influence the factor structure of the CoH-QoL-OQ scales, and further validation is advised in order to modify or even exclude some items from the Croatian version. The CoH-QoL-OQ in its original form, however, seems to show more favorable than unfavorable psychometric properties. 

Less than perfect model fit can be due to a specific sample. This sample included a substantial proportion of patients that have had a stoma for a long period of time, which can affect evaluation of their QoL because it is reasonable to assume new ostomy patients have most adaptation problems and issues affecting their QoL. For example, removing the item *Leaking pouch* seemed to improve the model fit, but it is the most frequent challenge that new ostomy patients face. Importance of this item is clinically justified, and it should not be removed from the questionnaire. Additionally, given a very good reliability and temporal stability, CoH-QoL-OQ can be used in research on Croatian patients for building the knowledge base about its use. Comparison of results with other countries is an additional reason that supports retaining all items in the original form of the questionnaire. 

## 5. Conclusions

City of Hope Quality of Life Ostomy Questionnaire is a reliable and valid questionnaire for the HRQoL assessment of the population of the Republic of Croatia. Although CFA has not fully confirmed that the data correspond to the established theoretical model, we advise using the original version of CoH-QoL-OQ among Croatian patients to preserve data comparability with other authors.

Regarding professional contribution, the study confirms that the CoH-QoL-OQ can be a useful tool for assessing health-related quality of life of ostomy patients. The use of CoH-QoL-OQ would enable healthcare professionals to conduct research and improve the QoL of patients in Croatia through individualized approach and better understanding of the needs of persons with ostomy.

## Figures and Tables

**Table 1:** Number and percentage of patients by sex, age, type of ostomy, duration of ostomy, and marital status, and mean and standard deviation for age and duration.

		*n*	(%)
Sex	Male	182	(60.3)
	Female	120	(39.7)
Total		302	(100.0)
Age groups	19–30 yrs	18	(6.0)
	31–50 yrs	68	(22.5)
	51–70 yrs	138	(45.7)
	70+ yrs	78	(25.8)
Total		302	(100.0)
Age (years)		59.28 ± 15.26	
Type of ostomy you have	Ileostomy	75	(24.8)
	Colostomy	221	(73.2)
	Urostomy	6	(2.0)
Total		302	(100.0)
If you have colostomy, is it permanent?	Permanent	212	(74.6)
	Temporary	72	(25.4)
Total		284	(100.0)
Do you wear the pouch all the time?	Yes	5	(25.0)
	No	15	(75.0)
Total		20	(100.0)
Duration of ostomy	Up to 6 months	60	(19.9)
	6–12 months	68	(22.5)
	1.1–2 years	26	(8.6)
	2.1–4 years	44	(14.6)
	4.1 + 8 years	38	(12.6)
	> 8 years	66	(21.9)
Total		302	(100.0)
Duration of ostomy (months)		55.23 ± 72.15	
What is your marital status	Single	45	(14.9)
	Married	198	(65.6)
	Divorced	27	(8.9)
	Widower/Widow	32	(10.6)
Total		302	(100.0)

**Table 2:** Descriptive indicators for the City of Hope Quality of Life-Ostomy Questionnaire (CoH-QoL-OQ) and subscales, and coefficients supporting very good Cronbach alpha internal consistency coefficient and test-retest reliability.

	Mean	SD	Median	1st Quartile	3rd Quartile	Minimum	Maximum	Valid N	N of Items	Cronbach Alpha	Test- Retest (*n* = 50)
Physical	6.85	1.82	7.00	5.54	7.00	1.82	10.00	N = 299	11	0.89	0.99
Psychological	6.24	2.03	6.15	4.92	6.15	0.31	10.00	N = 295	13	0.89	0.99
Social	6.03	1.89	6.00	4.75	6.00	0.83	10.00	N = 295	12	0.83	0.99
Spiritual	6.27	1.94	6.14	5.00	6.14	0.00	10.00	N = 298	7	0.73	0.99
COH Total	6.37	1.66	6.35	5.21	6.34	1.51	9.65	N = 284	43	0.95	0.99

**Table 3:** Convergent and discriminant validity indicators.

	Convergent Validity *	Discriminant Validity †			
		Physical	Psychological	Social	Spiritual
Physical	0.44–0.72	--	0.35–0.56	0.33–0.57	0.14–0.34
Psychological	0.33–0.69	0.22–0.60	--	0.30–0.66	0.28–0.62
Social	0.11–0.78	0.07–0.59	0.14–0.70	--	0.04–0.49
Spiritual	0.22–0.63	0.09–0.46	0.11–0.57	0.15–0.64	--

* Pearson coefficient of correlation of the items of a particular subscale with the total subscale score. † Pearson coefficient of correlation of the items of a particular subscale with the total score of other subscales.

**Table 4:** Item-total correlation with total COH and each subscale.

	Corrected Item-Total Correlation Total COH	Cronbach’s Alpha if Item Deleted Total COH	Physical	Psychological	Social	Spiritual
Physical strength (r)	0.542	0.945		0.437 **	0.493 **	0.208 **
Fatigue (r)	0.611	0.945		0.506 **	0.541 **	0.272 **
Skin surrounding the ostomy (r)	0.445	0.946		0.347 **	0.376 **	0.280 **
Sleep disorders (r)	0.656	0.944		0.562 **	0.574 **	0.342 **
Aches or pains (r)	0.614	0.945		0.525 **	0.483 **	0.273 **
Gas (r)	0.473	0.945		0.359 **	0.373 **	0.185 **
Odor (r)	0.537	0.945		0.472 **	0.441 **	0.259 **
Constipation (r)	0.429	0.946		0.360 **	0.330 **	0.221 **
Diarrhea (r)	0.509	0.945		0.451 **	0.413 **	0.251 **
Leaking from the pouch (or around the appliance) (r)	0.491	0.945		0.422 **	0.391 **	0.313 **
Overall physical well-being (r)	0.583	0.945		0.499 **	0.486 **	0.336 **
How difficult has it been for you to adjust to your ostomy? (r)	0.638	0.944		0.437 **	0.493 **	0.208 **
How useful do you feel?	0.414	0.946	0.592 **		0.602 **	0.402 **
How much satisfaction or enjoyment in life do you feel?	0.489	0.945	0.220 **		0.331 **	0.381 **
How much are you embarrassed by your ostomy? (r)	0.617	0.944	0.255 **		0.381 **	0.447 **
How good is your overall quality of life?	0.626	0.944	0.442 **		0.615 **	0.387 **
How is your ability to remember things?	0.339	0.946	0.413 **		0.539 **	0.468 **
How difficult is it to look at your ostomy? (r)	0.640	0.944	0.258 **		0.304 **	0.280 **
How difficult is it for you to care for your ostomy? (r)	0.669	0.944	0.439 **		0.588 **	0.416 **
Do you feel like you are in control of things in your life?	0.636	0.944	0.513 **		0.614 **	0.423 **
How satisfied are you with your appearance?	0.619	0.944	0.408 **		0.540 **	0.619 **
How much anxiety do you have? (r)	0.687	0.944	0.379 **		0.510 **	0.517 **
How much depression do you have? (r)	0.730	0.944	0.600 **		0.604 **	0.417 **
Are you fearful that your disease will come back? (r)	0.585	0.945	0.486 **		0.664 **	0.564 **
Do you have difficulty meeting new people? (r)	0.714	0.944	0.541 **	0.702 **		0.448 **
How much financial burden resulted from your illness or treatment? (r)	0.574	0.945	0.499 **	0.505 **		0.283 **
How distressing has your illness been for your family? (r)	0.244	0.947	0.302 **	0.191 **		0.036
How much does your ostomy interfere with your ability to travel? (r)	0.605	0.944	0.465 **	0.542 **		0.342 **
Has your ostomy interfered with your personal relationships? (r)	0.762	0.943	0.593 **	0.695 **		0.455 **
How much isolation is caused by your ostomy? (r)	0.733	0.943	0.491 **	0.711 **		0.489 **
Is support from friends and family sufficient to meet your needs?	0.181	0.947	0.045	0.204 **		0.317 **
Has your ostomy interfered with your recreational/sports activities? (r)	0.571	0.945	0.447 **	0.483 **		0.358 **
Has your ostomy interfered with your social activities? (r)	0.740	0.943	0.501 **	0.667 **		0.524 **
Has your ostomy interfered with your ability to be intimate? (r)	0.591	0.945	0.413 **	0.543 **		0.410 **
Do you have enough privacy at home for doing your ostomy care?	0.181	0.947	0.068	0.186 **		0.341 **
Do you have enough privacy when traveling for conducting your ostomy care?	0.180	0.947	0.107	0.141 *		0.256 **
How much uncertainty do you feel about your future? (r)	0.612	0.944	0.465 **	0.561 **	0.641 **	
Do you sense a reason for being alive?	0.425	0.946	0.206 **	0.440 **	0.378 **	
Do you have a sense of inner peace?	0.560	0.945	0.332 **	0.566 **	0.476 **	
How hopeful do you feel?	0.554	0.945	0.237 **	0.568 **	0.504 **	
Is support you receive from personal spiritual activities such as prayer or mediation sufficient to meet your needs?	0.297	0.947	0.165 **	0.278 **	0.208 **	
Is support you receive from religious activities such as going to church or synagogue sufficient to meet your needs?	0.171	0.948	0.088	0.110	0.146 *	
Has having an ostomy made positive changes in your lifestyle?	0.390	0.946	0.236 **	0.408 **	0.324 **	

* *p* < 0.05, ** *p*< 0.01.
